# Early diagnosis and risk factors of diabetic peripheral neuropathy in type 1 diabetes: insights from current perception threshold testing

**DOI:** 10.3389/fendo.2025.1496635

**Published:** 2025-03-31

**Authors:** Qian Zhao, Jialin Wang, Fangfang Liu, Hongwei Jiang, Yujin Ma

**Affiliations:** ^1^ Endocrinology and Metabolism Center, The First Affiliated Hospital, and College of Clinical Medicine of Henan University of Science and Technology, Luoyang, China; ^2^ Luoyang Key Laboratory of Clinical Multiomics and Translational Medicine, Henan Key Laboratory of Rare Diseases, Luoyang, China

**Keywords:** type 1 diabetes, diabetic peripheral neuropathy, current perception threshold, nerve conduction velocity, risk factors

## Abstract

**Objective:**

This study investigates nerve fiber dysfunction in type 1 diabetes (T1D) patients and identifies risk factors for diabetic peripheral neuropathy (DPN). It evaluates the relationship between current perception threshold (CPT) tests and nerve conduction velocity (NCV), and assesses CPT’s diagnostic accuracy for early DPN detection.

**Research design and methods:**

This study enrolled 110 patients with T1D and 26 healthy controls between January 2020 and December 2021, in accordance with predefined inclusion/exclusion criteria. CPT testing at 2000 Hz, 250 Hz, and 5 Hz assessed Aβ, Aδ, and C fiber function, while NCV was measured in 47 patients. Subgroups were stratified by disease duration (>5 years vs ≤5 years). Multivariate logistic regression identified DPN risk factors, and CPT-NCV correlation was analyzed using Chi-square and Kappa tests. Receiver operating characteristic (ROC) curves evaluated CPT diagnostic efficacy.

**Results:**

The incidence of DPN in 110 T1D patients was 78%, with no significant difference between disease duration subgroups (78.3% vs. 78.0%). Neurological abnormalities were significantly more common in the lower extremities compared to the upper extremities (67.27% vs. 49.09%, *P* < 0.05). Multivariate logistic regression analysis revealed that a waist-to-hip ratio (WHR) greater than 0.85 was an independent risk factor for DPN (*OR* = 3.01, 95% *CI*: 1.03-8.80, *P* < 0.05). Patients with a disease duration >5 years demonstrated significantly higher 2000Hz abnormality rates (68.09% vs. 46.15%, *P* < 0.05) and more severe neurological lesions (57.45% vs. 35.90%, *P* < 0.05). In contrast, those with disease duration ≤5 years exhibited elevated 5Hz abnormality rates (30.77% vs. 10.64%, *P* < 0.05) with predominantly milder lesions (56.41% vs. 31.91%, *P* < 0.05). Statistical analyses demonstrated a significant association between CPT and NCV (*P*<0.001), with moderate diagnostic consistency further supported by Cohen’s Kappa Test (κ=0.515, *P*<0.001). ROC curve analysis demonstrated that CPT exhibited moderate diagnostic accuracy in detecting DPN at the 5Hz, with an area under the curve (AUC) of 0.723.

**Conclusions:**

CPT showed moderate diagnostic accuracy for early unmyelinated (C) fibers detection, routine CPT screening in high-risk groups (central obesity/short disease duration) enables timely intervention to prevent irreversible damage.

## 1 Introduction

DPN is a common complication of T1D, characterized by its insidious onset, slow progression, rapid worsening, and irreversibility, affecting up to 50% of patients with diabetes (1). As demonstrated by some trials, DPN often is not present at the moment of diagnosis, while it could appear after at least 10 years of disease duration, and could affect up to 34% of subjects after ~25 years (2). Therefore, early diagnosis and intervention are essential to prevent these outcomes. In the early stages of diabetic neuropathy or upon a pre-diabetes diagnosis, patients typically experience small fiber neuropathy (3), characterized by distal pain symptoms such as burning, stabbing, and freezing sensations, which tend to worsen during periods of rest. Large fiber damage generally manifests later in the disease progression. Currently, the diagnosis of DPN primarily relies on clinical symptoms and NCV examination, which remains a key diagnostic method. However, NCV is normal in patients with mainly small fiber neuropathy, and clinical tests in these patients are usually almost normal (4). Recent research has highlighted the significance of small nerve fibers, as they appear to be critical targets for the early detection of diabetic peripheral neuropathy. Some studies suggest that small nerve fibers may exhibit detectable damage years before large nerve fibers are affected (5). The gold standard for diagnosing small fiber neuropathy is the measurement of intraepidermal nerve fiber density (IENFD) by skin needle biopsy (6), but this invasive method is rarely needed in routine diagnosis. CPT as an innovative technique for the comprehensive and quantitative assessment of sensory nerve fiber function, demonstrates excellent reliability and stability (7). Unlike traditional neurophysiological methods, which can only detect damage to coarsely myelinated (Aβ) fibers, CPT allows for the assessment of finely myelinated (Aδ) fibers and unmyelinated (C) fibers by adjusting the stimulation frequency (7). Studies (8) have shown that CPT abnormalities can occur before changes in NCV, suggesting that CPT may provide a more sensitive and earlier diagnostic method for detecting DPN. This study aimed to evaluate the diagnostic utility of CPT and identifying key risk factors for DPN, explore whether CPT is more sensitive than NCV in the early detection of diabetic peripheral neuropathy in T1D patients. By we provide evidence for the use of CPT as a routine screening tool for early detection of DPN in clinical practice.

## 2 Materials and methods

### 2.1 Patient selection

This study included 110 confirmed T1D patients and 26 healthy subjects who were treated at the Department of Endocrinology, The First Affiliated Hospital of Henan University of Science and Technology, from January 2020 to December 2021. The inclusion of healthy controls in this study was essential for establishing baseline values of the CPT measures, which serve as a reference for identifying abnormalities in patients with T1D. The comparison with healthy controls allows for a more accurate interpretation of the data and helps to establish the degree of nerve dysfunction in patients with DPN relative to individuals without diabetes. All patients and their families were informed about the study and voluntarily agreed to participate. The study was approved by the Ethics Committee of The First Affiliated Hospital of Henan University of Science and Technology (approval number 2022-03-B066).

#### 2.1.1 Inclusion criteria

Inclusion of T1D: (1) T1D patients (all age groups), in line with the ADA diagnostic criteria for diabetes (9); (2) None of the patients received drug therapy that affected neurological function, including but not limited to: Antiepileptic drugs (e.g., carbamazepine, phenytoin), Antidepressants (e.g., amitriptyline, duloxetine), Antipsychotics (e.g., haloperidol, risperidone), Chemotherapeutic agents (e.g., vincristine, cisplatin), Other neurotoxic drugs (e.g., isoniazid); (3) Voluntary participation in this study.

Inclusion of Healthy Control: The Healthy Control group consisting of individuals who are free from specific conditions or diseases and are used for comparison in our study.

#### 2.1.2 Exclusion criteria

(1) Type 2 diabetes (T2D), gestational diabetes, special types of diabetes; (2) Any serious disease or condition that may affect the subject’s ability to complete the study or compromise patient safety (such as malignancies, end-stage organ failure, or acute infections—health risks that could jeopardize patient well-being or data validity, economic factors (e.g., treatment expenses) were not the exclusion rationale); (3) Combined with neuropathy caused by other reasons: including toxins (e.g., alcohol), neurotoxic medications (e.g., chemotherapy), vitamin B12 deficiency, hypothyroidism, renal disease, malignancies (e.g., multiple myeloma, bronchogenic carcinoma), infections (e.g., HIV), chronic inflammatory demyelinating neuropathy, inherited neuropathies, and vasculitis (9); (4) Primary glomerular disease, acute kidney injury, urinary tract infection; (5) Subjects with conditions that, in the investigator’s judgment, would interfere with compliance, data integrity, or safety during the study, such as severe cognitive impairment, communication barriers, or an inability to complete required assessments.

### 2.2 Data collection

#### 2.2.1 Clinical characteristics

The patient’s age, course of disease, gender, place of residence, educational level, height, weight, body mass index (BMI), waist circumference, hip circumference, WHR, daily insulin dose, and insulin pump usage were recorded.

#### 2.2.2 Laboratory index

All subjects fasted for more than 8 hours, and HbA1c, cholesterol, triglyceride, high-density lipoprotein (HDL), low-density lipoprotein (LDL), creatinine, blood urea nitrogen (BUN), estimated glomerular filtration rate (eGFR), uric acid, urine microalbumin, and urinary albumin-creatinine ratio (UACR) were detected in the next morning; The non-mydriatic fundus examination and visceral fat detection were performed.

#### 2.2.3 Sensory nerve function testing and nerve conduction testing

Neurometer. CPT/C sensory nerve quantitative detector was used to detect sensory nerve function, and CPT was used to measure the bilateral median nerve and sural nerve function with 3 predetermined frequencies (5 Hz, 250 Hz and 2000 Hz). The normal value range of CPT is: The median nerve was 291.4 ± 81.8mA×100 at 2000Hz, 102.0 ± 60.0mA×100 at 250Hz, and 60.0 ± 34.2mA×100 at 5Hz; The sural nerve was 375.0 ± 120.5mA×100 at 2000Hz, 136.0 ± 52.3mA×100 at 250Hz, and 92.3 ± 54.4mA×100 at 5Hz. Those within the normal range are considered normal, below the normal value are hyperesthesia, and above the normal value are hypoesthesia. According to the paresthesia at 2000Hz, 250Hz and 5Hz, the damage of Aβ, Aδ and C nerve fibers was obtained, and graded as mild, moderate and severe.

EMG was used to measure NCV in patients with T1D, including sensory nerve conduction velocity and motor nerve conduction velocity. Abnormal conduction velocity can be defined based on the following thresholds: (1) Motor Nerve Conduction Velocity (Motor NCV):Median nerve: ≥48 m/s, Ulnar nerve: ≥48 m/s, Common peroneal nerve: ≥44 m/s, Tibial nerve: ≥42 m/s; (2) Sensory Nerve Conduction Velocity (Sensory NCV): Median nerve: ≥45 m/s, Ulnar nerve: ≥44.2 m/s, Superficial peroneal nerve: ≥45.4 m/s.

### 2.3 Statistical analysis

Statistical data analysis was performed using SPSS 26.0 software. For normally distributed measurement data, a t-test was used for group comparisons. Non-normally distributed data were expressed as the median (interquartile range) [*P50 (P25, P75)*], and a nonparametric test was used for group comparisons. Categorical data were presented as percentages (%), and the chi-square test was used for group comparisons. Univariate and multivariate logistic regression analyses were conducted to identify the risk factors for abnormal CPT in T1D patients, and the OR with its 95% confidence interval was calculated. The association between NCV and CPT diagnoses was statistically assessed using the chi-square test, while inter-rater agreement was evaluated through the Cohen’s Kappa Test. To assess the diagnostic potential of CPT for DPN, the AUC was calculated using ROC curve analysis to evaluate the diagnostic performance of CPT. The significance level was set at α = 0.05, with *P* < 0.05 considered statistically significant.

## 3 Result

### 3.1 Baseline data of T1D group

Among the 110 T1D patients, 54 were male and 56 were female, with an average age of 23 years (range: 14–36) and an average disease duration of 6 years (range: 2–9). The HbA1c level was 8.45% (range: 7.10%–10.92%), with 48 patients (43.6%) using insulin pumps. The incidence of DR was 20.9%, DKD was 24.5%, and DPN was 78% (**Supplementary Table S1**). The normal control group consisted of 26 individuals, 11 males and 15 females, with an average age of 23 years (range: 20–26).

A total of 110 T1D patients were categorized into two groups based on disease duration: >5 years (n=60) and ≤5 years (n=50). Compared to patients with a disease duration of ≤5 years, those with a disease duration of >5 years exhibited significantly higher levels of body weight, BMI, total daily insulin, LDL, triglycerides, cholesterol, creatinine, uric acid, and UACR, while eGFR showed a declining trend. These differences were statistically significant (*P* < 0.05). Additionally, the incidence of DR and DKD increased with disease duration, and the differences were significant (*P* < 0.05). However, no significant differences were observed between the groups in terms of WHR, HbA1c, insulin pump usage, HDL, BUN, urinary microalbumin, visceral fat area, DPN prevalence, symptomatic DPN prevalence, or the incidence of hypoglycemia events (*P* > 0.05) (**Supplementary Table S1**).

### 3.2 Comparison of CPT values at different current frequencies between T1D group and normal control group

Both the T1D group and the normal control group were tested using a current sensory nerve measuring instrument, with CPT values measured at 2000Hz, 250Hz, and 5Hz stimulation frequencies (**Supplementary Table S2**). The CPT values for the bilateral median and sural nerves in the T1D group were higher than those in the normal control group, and the difference was statistically significant (*P* < 0.05).

### 3.3 Analysis of abnormal rate of upper and lower extremity nerves in T1D patients

The abnormal rate of the median nerve was 49.09% (54/110), while the abnormal rate of the sural nerve was 67.27% (74/110). A paired chi-square test was used to compare the abnormal rates of peripheral nerves in the upper and lower extremities. The results showed that the peripheral nerves of the lower extremities were more vulnerable than those of the upper extremities in patients with T1D (*P* = 0.009, **Table 1**).

**Table 1:** Analysis of abnormal rate of upper and lower extremity nerves in T1D patients.

		Median nerve		Total
		normal	abnormal	
Sural nerve	normal	19	17	36
	abnormal	37	37	74
Total		56	54	110

The abnormalities of upper limb median nerve and lower limb sural nerve in T1D patients were detected by CPT, and the abnormal rates of upper and lower limbs were compared and analyzed, and the vulnerable limbs were evaluated.

### 3.4 Risk factor analysis of DPN

DPN was set as the dependent variable, and univariate logistic regression analysis was performed using the independent variables of age, disease duration, body weight, BMI, waist-to-hip ratio, HbA1c, number of insulin pump users, daily insulin dose, cholesterol, triglycerides, HDL, LDL, creatinine, BUN, eGFR, uric acid, urine microalbumin, ACR, visceral fat area, DR, DKD, symptomatic DPN, and hypoglycemic events. The analysis revealed that waist-to-hip ratio and DKD were significantly correlated with the occurrence of DPN (*P* < 0.05). Patients with a WHR > 0.85 were grouped separately and included in a multivariate logistic regression analysis with WHR and DKD. It was found that a WHR > 0.85 was an independent risk factor for DPN (*P* < 0.05), with an OR of 3.01 (95% *CI*: 1.03–8.80) (**Table 2**).

**Table 2:** Multivariate logistic regression analysis results of DPN.

Variable	Partial regression coefficient β	Standard error	Wald value	*P* value	*OR* value	95% *CI*
Constant	-0.18	0.72	0.06	0.81		
Waist-to-hip ratio (>0.85)	1.10	0.55	4.05	0.04	3.01	(1.03, 8.80)

The occurrence of waist-to-hip ratio > 0.85 was a risk factor for DPN (P < 0.05), and the OR value was 3.01 (95%CI 1.03-8.80), indicating that the risk of DPN in patients with waist-to-hip ratio > 0.85 was 3.01 times that in patients with waist-to-hip ratio < 0.85.

### 3.5 Comparison of abnormal rate and lesion degree of T1D patients with abnormal CPT in different course conditions

Among 110 T1D patients, 86 patients had abnormal CPT detection. 86 patients with abnormal CPT were grouped based on disease duration: > 5 years and ≤ 5 years. The abnormal rates of CPT between the two groups were compared, and the results showed that the abnormal rates for the 2000Hz and 5Hz frequencies in patients with disease duration > 5 years were 68.09% and 10.64%, respectively. In patients with disease duration ≤ 5 years, the abnormal rates for the 2000Hz and 5Hz frequencies were 46.15% and 30.77%, respectively. A significant difference was found between the two groups (*P* < 0.05). However, no significant difference was observed at the 250Hz frequency (*P* > 0.05) (**Table 3**).

**Table 3:** Comparison of abnormal rates of patients with abnormal CPT in different course groups under three current frequencies.

		Disease duration ≤ 5 years (n=39)	Disease duration>5 years (n=47)	z/χ2	*P* value
2000Hz	n, %	18, 46.15%	32, 68.09%	4.212	0.040
250Hz	n, %	9, 23.08%	10, 21.27%	0.009	0.926
5Hz	n, %	12, 30.77%	5, 10.64%	5.446	0.020

The 86 patients with abnormal CPT were categorized according to the severity of their lesions (mild, moderate, and severe). The abnormal rates of each lesion severity were compared between the two groups. The results showed that the incidence of severe lesions was higher in patients with disease duration > 5 years, while the incidence of mild lesions was higher in patients with disease duration ≤ 5 years. This difference was statistically significant (*P* < 0.05). No significant difference was found in the incidence of moderate lesions between the two groups (*P* > 0.05) (**Table 4**).

**Table 4:** Comparison of lesion severity in patients with abnormal CPT in different course groups.

	Disease duration ≤ 5 years (n=39)	Disease duration > 5 years (n=47)	χ2	*P* value
Mild lesions (n, %)	22, 56.41%	15, 31.91%	5.217	0.022
Moderate lesions (n, %)	3, 7.69%	5, 10.64%	0.423	0.515
Severe lesions (n, %)	14, 35.90%	27, 57.45%	3.968	0.046

### 3.6 Correlation analysis between CPT values and NCV results in TIDM patients

Among the 110 T1D patients, 47 underwent NCV testing and were divided into two groups based on the test results: the abnormal NCV group (25 patients) and the normal NCV group (22 patients). Due to the symmetry and distal distribution of DPN, CPT test data for the right lower extremity sural nerve at different frequencies (2000 Hz, 250 Hz, and 5 Hz) were selected. The chi-square test identified a significant association between NCV and CPT diagnoses (*P*<0.001; **Table 5**). CPT exhibited perfect sensitivity (100%) but limited specificity (50%) for detection. Cohen’s Kappa Test further supported moderate diagnostic consistency (κ = 0.515, *P*<0.001; **Table 6**).

**Table 5:** Correlation analysis of CPT and NCV.

		NCV		Total
		normal	abnormal	
CPT	normal	11	0	11
	abnormal	11	25	36
Total		22	25	47

The chi-square test identified a significant association between NCV and CPT diagnoses (*P*<0.001). According to the data in the four-fold table, the sensitivity of CPT was 100% and the specificity was 50%.

**Table 6:** Cohen’s Kappa test for the CPT and the NCV.

		value (κ)	Asymptotic standard error	Approximate T	*P*
Protocol variable	Kappa	0.515	0.112	4.040	0.000

The interpretation criteria for Kappa values (κ) are as follows: κ ≤ 0 indicates no agreement, 0 < κ ≤ 0.2 indicates slight agreement, 0.2 < κ ≤ 0.4 indicates fair agreement, 0.4 < κ ≤ 0.6 indicates moderate agreement, 0.6 < κ ≤ 0.8 indicates substantial agreement, and κ > 0.8 indicates almost perfect agreement.

**Figure 1:**
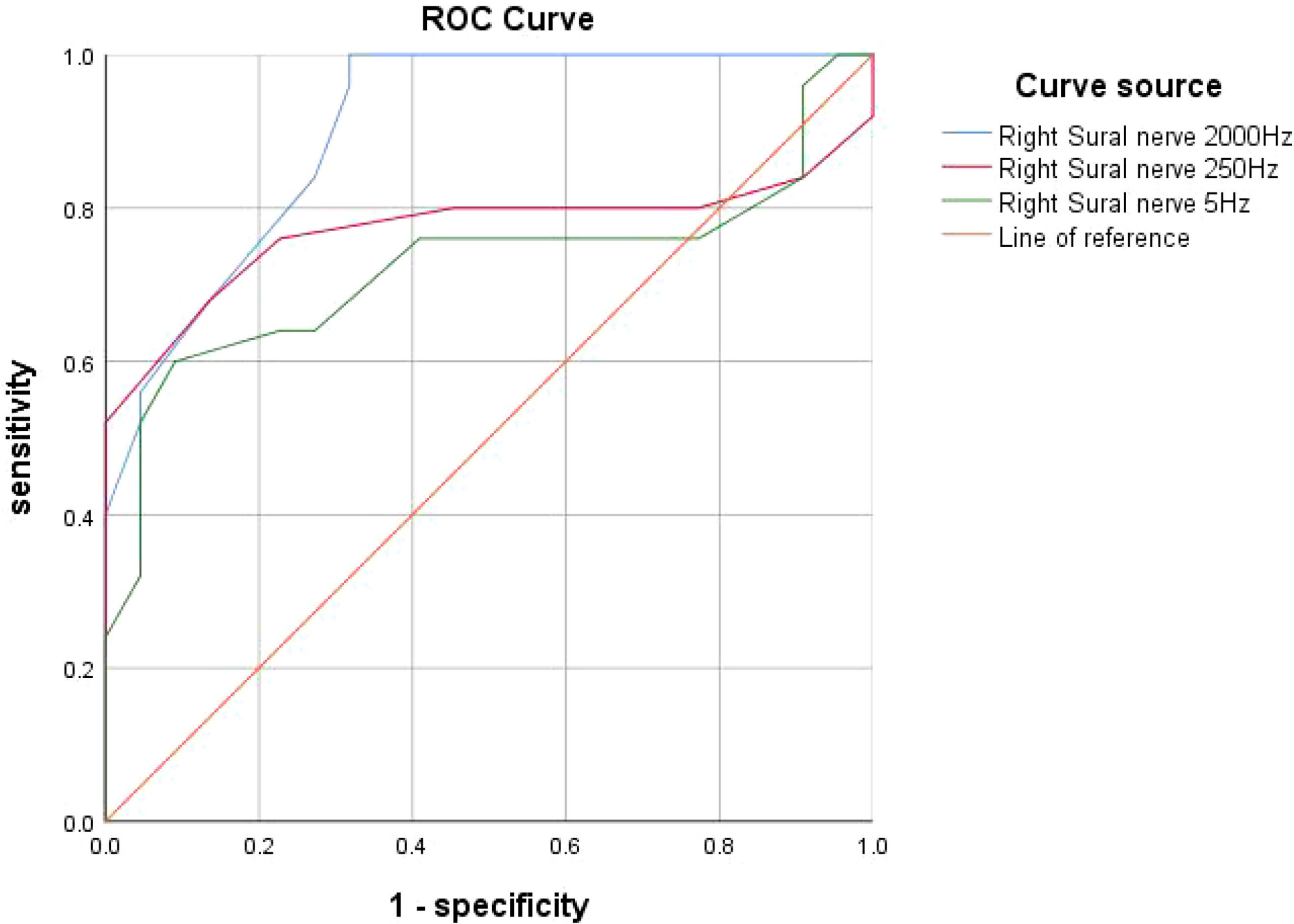
ROC curve: Diagnostic performance of CPT for diabetic peripheral neuropathy at different frequencies. ROC curve for CPT test compared with NCS: the AUC for the right sural nerve at 2000 Hz was 0.904 (*P* < 0.001). The AUC for the right sural nerve at 250 Hz was 0.771 (*P* = 0.001). The AUC for the right sural nerve at 5 Hz was 0.723 (*P* < 0.05).

The AUC was calculated through ROC curve analysis to assess the diagnostic performance of CPT. The results showed that the AUC for the right sural nerve at 2000 Hz was 0.904 (*P* < 0.001, 95% *CI:* 0.82-0.99). The AUC for the right sural nerve at 250 Hz was 0.771 (*P* = 0.001, 95% *CI*: 0.62-0.92). The AUC for the right sural nerve at 5 Hz was 0.723 (*P* = 0.009, 95% *CI*: 0.57-0.88) (**Figure 1**).

## 4 Discussions

DPN is the most common chronic complication in patients with T1D, yet its pathogenesis remains unclear. Studies have shown that hyperglycemia-induced oxidative stress may lead to metabolic disorders and microvascular damage, which are believed to contribute significantly to the development of DPN (1). The ADA guidelines recommend that individuals with T1D for ≥ 5 years and all individuals with T2D should undergo annual DPN evaluation (9).The evaluation of DPN begins with a thorough neurological and medical history, followed by electrodiagnostic testing (9). Symptoms vary depending on the type of sensory fibers affected. Involvement of large fibers can result in numbness and a loss of protective sensation, while the most common early symptoms, such as pain and numbness (characterized by unpleasant burning and tingling sensations), are typically caused by small fiber involvement (10). Currently, DPN screening primarily focuses on diagnosing the loss of protective sensation, which involves testing only for large fiber function. However, small fiber neuropathy, which is often involved in the early stages of DPN, remains challenging to detect reliably (11). Therefore, improving the detection of peripheral small fiber nerve injury is essential for early DPN diagnosis.

Skin biopsy, which measures the density of epidermal nerve fibers, is considered the gold standard for diagnosing small fiber neuropathy. Its specificity can reach 95%, and its sensitivity is 90% (12). Skin biopsy is particularly useful in patients with clinical manifestations of peripheral neuropathy but no positive electrophysiological findings (12). However, it is associated with relatively high costs, cannot be repeated on the same skin area, is time-consuming, requires histological laboratories, and may lead to infection and bleeding (13). Corneal confocal microscopy (CCM) is a novel, rapid, non-invasive, reproducible, and quantifiable method for detecting small neuropathies (14). Automated analysis and standardized evaluation criteria make it suitable for clinical application (15). CCM allows repeated analysis of the same corneal section, enabling longitudinal assessments of disease progression and treatment effects. It can also visualize dendritic cell morphology and density, providing insights into the severity of underlying autoimmune and inflammatory processes (16, 17). However, CCM requires specialized equipment and trained professionals, which can limit its clinical application.

### 4.1 CPT as a sensitive tool for early DPN detection

As a quantitative sensory examination technique, CPT is non-invasive, neuroselective, and an effective means for comprehensive assessment of peripheral neuropathy. CPT induces nerve impulses in Aβ, Aδ, and C fibers through 2000 Hz, 250 Hz, and 5 Hz sinusoidal alternating current stimulation. The stimulation frequency can be adjusted to evaluate the function of different nerve fibers, providing valuable evidence for the early diagnosis of DPN (7). However, few studies have examined whether CPT can effectively detect early-stage DPN in T1D patients. This study assesses the incidence of DPN in T1D patients using CPT and compares it with NCV results to provide a basis for early clinical diagnosis of DPN. In a study on diabetes and CPT in children and adolescents (18), 92 T1D patients without neurological symptoms were assessed, with a mean age of 14.2 ± 2.1 years and a mean disease duration of 5.8 ± 3.0 years. Twenty-one (23%) diabetic children exhibited early sensory nerve dysfunction, and compared with the normal control group, the CPT values at 2000 Hz were higher. The results suggest that the CPT test can be used for detecting subclinical peripheral neuropathy in children and adolescents with diabetes. In our study, 26 healthy control participants were included, and no significant abnormalities were detected in this group using CPT. The CPT values at 2000 Hz, 250 Hz, and 5 Hz were significantly higher in patients with T1D compared to the control group. This further demonstrates that CPT can effectively distinguish between normal and abnormal nerve fibers. The most common form of DPN is length-dependent diabetic polyneuropathy (LDDP), which progresses in a length-dependent manner, starting from the feet and advancing to the proximal lower limbs, then to the distal upper limbs, and eventually to the proximal upper limbs (19). In this study, we compared and analyzed the upper and lower extremities of patients and found that the abnormal rate in the lower extremities was higher than that in the upper extremities. A similar phenomenon was also observed by Olsen et al. (20).

DPN accounts for approximately 75% of all diabetic neuropathies, affecting up to 50% of patients with T1DM and T2DM, as well as at least 10% of individuals with prediabetes. However, the estimated prevalence exhibits heterogeneity and wide variability, depending on the diagnostic methods applied (21). A study evaluating the prevalence of DPN in T1D patients (22) found that the prevalence of DPN varies widely (approximately 8%-54%) due to different etiological and/or methodological factors. In a multicenter study involving 1,113 diabetic patients in Turkey, the prevalence of DPN was found to be 40.4% based on clinical examination alone. However, when nerve conduction studies were combined with clinical examination, the prevalence of DPN increased to 62.2%, and the prevalence of neuropathic pain was 14.0% (23). In another study from Turkey, screening 100 newly diagnosed pre-diabetic individuals for microvascular and macrovascular diabetes complications revealed that 12% of participants had microvascular complications (neuropathy: 4%, kidney disease: 8%), while 19% had macrovascular complications (24). The Michigan Neuropathy Screening Instrument Questionnaire (MNSIQ) was used to assess the prevalence of DPN in T1D patients with a disease duration of ≥5 years., the prevalence of DPN in 5,936 T1D patients was only 11% (25). Another study using MNSIQ to assess the prevalence of DPN in 5,558 T1D patients found that the overall prevalence of DPN was 13% (26). In this study, the median age of 110 T1DM patients was 23 years old and the median course of disease was 6 years. The prevalence rate of DPN was 78% by CPT test. There was no significant difference in the prevalence of DPN between patients with a disease course > 5 years and those with a disease course ≤ 5 years (78.30% vs. 78.00%). The 86 patients with abnormal CPT were further divided into two groups based on disease course (> 5 years and ≤ 5 years). At the 5Hz frequency, the abnormal rate and incidence of mild neuropathy were higher in patients with a disease course ≤ 5 years (30.77% vs. 10.64%, 57.45% vs. 35.90%). However, at the 2000Hz frequency, patients with a disease course > 5 years had a higher incidence of abnormality and severe neuropathy (68.09% vs. 46.15%, 56.41% vs. 31.91%). These results suggest that because CPT can evaluate small fiber nerve injury through 5Hz current stimulation, mild small fiber injury is more frequently detected in the early stages of diabetes, leading to no significant difference in DPN prevalence when grouped by disease course. As the disease progresses, neuropathy gradually worsens, and functional impairment of large fibers, detectable by 2000Hz current stimulation, becomes more prominent.

Correlation analysis revealed a statistically significant association between CPT and NCV, with high sensitivity. Consistency testing indicated moderate agreement between NCV and CPT. These findings suggest that CPT has diagnostic capability for DPN compared to NCV. The high sensitivity implies that all true positive cases were correctly identified (no missed diagnoses), while the specificity of 50% indicates a potential issue with false positives, which may lead to overmedicalization or resource waste (e.g., unnecessary follow-up tests or treatments). We calculated the AUC using ROC curve analysis to evaluate the diagnostic performance of CPT compared to NCV. The analysis revealed significant differences in diagnostic performance across different frequencies. The 2000 Hz frequency demonstrated the best diagnostic accuracy (AUC=0.904), while the 250 Hz and 5 Hz frequencies showed moderate diagnostic accuracy (AUC=0.771 and AUC=0.723, respectively). Therefore, we propose that CPT can serve as a complementary diagnostic tool to NCV, particularly for assessing small fiber nerve function, demonstrating moderate diagnostic capability and high sensitivity. However, clinicians should be cautious about potential false positives during its application. To avoid misdiagnosis, CPT results should be interpreted in conjunction with patient symptoms and other clinical findings. While CPT evaluates small fiber nerve function, NCV primarily assesses large fiber nerve function. The combination of both methods enables earlier and more accurate diagnosis of DPN.

### 4.2 Risk factors for DPN

A longer duration of diabetes, elevated HbA1c levels, dyslipidemia, and the presence of comorbidities such as hypertension, cardiovascular disease, and kidney disease have been widely recognized as significant risk factors for DPN (27). A meta-analysis of risk factors for DPN showed that diabetes duration, age, HbA1c levels, and diabetic retinopathy (DR) were significantly associated with an increased risk of DPN (28). The DCCT/EDIC study indicated that a higher HbA1c level was the most significant risk factor for DPN, followed by older age, longer disease duration, increased height, macroalbuminuria, higher mean pulse rate, use of beta-blockers, and persistent albuminuria (29). In our study, we did not identify the same risk factors for DPN (such as disease course, HbA1c, and comorbidities) as those reported in previous studies. This discrepancy may be attributed to the fact that the diagnosis of DPN in our study primarily focused on small fiber neuropathy, whereas previous studies mainly concentrated on large fiber neuropathy. This difference in diagnostic focus could lead to variations in the observed risk factors.

A study examining the relationship between obesity and neuropathy found that waist circumference (OR = 1.39; 95% CI: 1.10-1.75) was significantly associated with neuropathy (30). Zhou et al. also suggested that a larger waist circumference was linked to a higher risk of DPN (31). Additionally, a study exploring the association between metabolic and lifestyle factors and DPN in patients with early type 2 diabetes showed that among 5,249 patients with a median disease duration of 2.8 years, 17.9% (n = 938) had DPN. Regression analysis revealed that central obesity (waist circumference, waist-to-hip ratio, and waist-to-height ratio) was significantly associated with DPN (32). Prospective observations and MR analyses also provide evidence that high BMI and waist circumference represent potential causative risk factors for DPN (33). These findings suggest that weight control may modify the risk of these complications independently of glycemic control. Our study also identified a waist-to-hip ratio > 0.85 as an independent risk factor for DPN (OR = 3.01; 95% CI: 1.03-8.80), this aligns with previous findings, underscoring the significance of central obesity in the development of DPN. These studies all suggest that obesity, particularly central obesity, is a significant risk factor for DPN. Weight control not only aids in blood sugar management but may also independently reduce the risk of neuropathy. Therefore, in clinical practice, in addition to focusing on blood sugar control, it is essential to emphasize weight management and the reduction of central obesity to prevent or delay the onset and progression of neuropathy.

### 4.3 Clinical application or further research implications

#### 4.3.1 CPT as a routine screening tool and patient education

Our results suggest that CPT testing should be considered as a routine screening method for DPN in T1D patients, particularly in those with a disease duration of <5 years or other risk factors such as central obesity. The ability of CPT to detect early-stage small fiber neuropathy, which is often asymptomatic, makes it a valuable complement to traditional diagnostic methods such as NCV testing. By identifying patients at risk of DPN before the onset of clinical symptoms, clinicians can implement targeted interventions to prevent disease progression and reduce the risk of severe complications such as diabetic foot ulcers and amputations. Given its non-invasive nature, reproducibility, and ease of administration, CPT testing is well-suited for integration into routine clinical practice. We recommend that healthcare providers consider incorporating CPT testing into annual diabetes check-ups for high-risk patients, particularly those with poor glycemic control, long disease duration, or elevated WHR. Early detection of DPN using CPT testing could prompt tighter glycemic control, lifestyle modifications, and the use of neuroprotective agents, all of which have been shown to slow the progression of DPN.

The identification of WHR >0.85 as a risk factor for DPN highlights the importance of addressing central obesity in diabetes management. Clinicians should consider incorporating WHR measurements into routine assessments and educate patients about the risks associated with central obesity. Lifestyle interventions aimed at reducing central obesity, such as weight loss and physical activity, may help mitigate the risk of DPN in T1D patients.

#### 4.3.2 Future research directions

While our findings are promising, further validation in larger, multicenter studies is needed to confirm the generalizability of our results. Future studies should include diverse populations with varying demographics, disease durations, and comorbidities to ensure the broad applicability of CPT testing. Longitudinal studies are needed to evaluate the long-term predictive value of CPT testing for DPN progression and its impact on clinical outcomes. The cost-effectiveness of CPT testing as a routine screening tool should be evaluated to determine its feasibility in different healthcare settings. This analysis could inform clinical guidelines and reimbursement policies, facilitating the widespread adoption of CPT testing.

## 5 Limitations

This study is a cross-sectional study with a small sample size, and the data may be biased. Since this study recruited participants from a single medical center, selection bias may exist. To reduce this, we implemented predefined inclusion and exclusion criteria to ensure a representative patient cohort. Future studies will consider multicenter validation to enhance generalizability. Variability in data collection could introduce bias. To minimize this, we standardized all assessments, trained study personnel, and employed validated diagnostic tools (e.g., CPT and NCV measurements followed a standardized protocol). Potential confounding factors such as age, disease duration, glycemic control, and obesity may affect DPN outcomes. To control for these, we performed multivariate logistic regression analysis to adjust for these variables and identify independent risk factors. Investigators conducting CPT and NCV assessments were blinded to patients’ clinical history to prevent subjective influence on data interpretation. Despite these limitations, this study provides valuable insights into the early detection of DPN in T1D patients and highlights key risk factors. Future research with larger, more diverse cohorts and longitudinal follow-up is warranted to further validate these findings.

## 6 Conclusion

In conclusion, our study supports the use of CPT testing as a sensitive and practical tool for the early diagnosis of DPN in T1D patients. The integration of CPT testing into routine clinical practice, along with targeted interventions for central obesity or a shorter disease duration, has the potential to improve outcomes and reduce the burden of diabetic complications. Future research should focus on validating these findings in larger, more diverse populations and exploring the long-term benefits of early DPN detection and intervention.

## Data Availability

The original contributions presented in the study are included in the article/**Supplementary Material**. Further inquiries can be directed to the corresponding authors.

## Supplementary material

Supplementary Table 1 Baseline clinical data of T1DM patients [(n, %), ( $\bar{x}\pm s$ ), *P50 (P25, P75)*]. (110 patients with T1DM were grouped according to disease duration > 5 years and ≤5 years, and the differences of various indicators between the two groups were compared, The significance level was set at α = 0.05, with P < 0.05 considered statistically significant.)

Supplementary Table 2 Comparison of CPT values between T1DM group and normal control group [*P50 (P25, P75)*]. A comparison with a healthy control group could interpret the data more accurately and help determine neurological dysfunction in DPN patients relative to non-diabetic patients, The significance level was set at α = 0.05, with P < 0.05 considered statistically significant.
