# Supplementary material for: Early diagnosis and risk factors of diabetic peripheral neuropathy in type 1 diabetes: insights from current perception threshold testing
Source: Front Endocrinol (Lausanne). 2025 Mar 31;16:1496635. doi: 10.3389/fendo.2025.1496635 (PMC11994408; doi:10.3389/fendo.2025.1496635)
Supplement: Supplementary Table 1 — Baseline clinical data of T1DM patients [(n, %), ( x¯±s ), P50 (P25, P75)]. (110 patients with T1DM were grouped according to disease duration > 5 years and ≤5 years, and the differences of various indicators between the two groups were compared, The significance level was set at α = 0.05, with P < 0.05 considered statistically significant.) [file Table1.docx]

Table S1 Baseline clinical data of T1DM patients [(n, %), ( $\bar{x}\pm s$ ), (P50 (P25, P75)]

(110 patients with T1DM were grouped according to disease duration > 5 years and ≤5 years, and the differences of various indicators between the two groups were compared, The significance level was set at α = 0.05, with P < 0.05 considered statistically significant.)

| Index | T1DM | Duration ≤5 years | Duration ＞5 years | *P* |
| --- | --- | --- | --- | --- |
| Number | 110 | 50 | 60 |  |
| Gender (male, female) | 54, 56 | 27 23 | 27 23 |  |
| Age(years) | 23（14,36） | 16.50(12.00, 32.25) | 27.00（18.00, 37.75） | 0.002 |
| Disease duration (years) | 6（2,9） | 2.00（1.00, 4.00） | 9.00（7.00, 15.00） | ＜0.001 |
| Weight (Kg) | 55.17±12.82 | 49.64±12.94 | 59.78±10.82 | ＜0.001 |
| BMI（Kg/m^2^） | 20.60±2.91 | 19.18±2.50 | 21.79±2,71 | ＜0.001 |
| Waist to hip ratio | 0.84（0.82, 0.88） | 0.84（0.83, 0.87） | 0.84（0.80, 0.89） | 0.688 |
| HbA1c（%） | 8.45（7.10, 10.92） | 8.80（6.50, 11.20） | 8.20（7.23, 10.38） | 0.978 |
| Total daily insulin dose (IU/day) | 40.00（27.90, 49.25） | 34.65±16.25 | 44.55±16.13 | 0.002 |
| Insulin pump user (n) | 48（43.6%） | 23.00（46.00%） | 25.00（41.70%） | 0.648 |
| Cholesterol (mmol/L) | 4.70（4.09, 4.97） | 4.44（3.94, 4.97） | 4.97（4.10, 4.97） | 0.013 |
| Triglycerides (mmol/L) | 1.20（0.71, 1.28） | 0.85（0.69, 1.23） | 1.23（0.82, 1.35） | 0.020 |
| HDL（mmol/L） | 1.45（1.27, 1.52） | 1.45（1.20, 1.63） | 1.45（1.29, 1.49） | 0.816 |
| LDL（mmol/L） | 2.48（2.14, 2.73） | 2.39（1.88, 2.52） | 2.48（2.27, 3.08） | 0.005 |
| BUN（mmol/L） | 5.47（4.45, 6.23） | 5.10（4.50, 6.03） | 5.47（4.37, 6.38） | 0.085 |
| Creatinine (mmol/L) | 58.02（45.25, 65.43） | 50.50（40.31, 61.99） | 58.02（50.08, 66.75） | 0.003 |
| eGFR（ml/min/1.73 m^2^） | 128.94±26.82 | 139.40±29.03 | 120.23±21.43 | ＜0.001 |
| Uric acid (umol/L) | 291.03（232.00, 321.25） | 270.50（225.50, 314.86） | 293.05（237.50, 321.75） | ＜0.001 |
| Urine microalbumin-creatinine ratio (㎎/g) | 12.46（7.03, 46.50） | 8.80（6.16, 25.08） | 14.95（8.20, 50.70） | 0.016 |
| Visceral fat area（cm^2^） | 47.00（35.75, 52.00） | 47.00（44.00, 47.00） | 47.00（32.25, 59.00） | 0.750 |
| Peripheral neuropathy (abnormal CPT) | 86（78.18%） | 39（78.00） | 47（78.30） | 0.966 |
| Diabetic retinopathy | 24（21.81%） | 3（6.00） | 20（33.30） | ＜0.001 |
| Diabetic nephropathy | 27（24.50%） | 3（6.00） | 24（40.00） | ＜0.001 |
| Hypoglycemia event | 71（64.50%） | 30（60.00） | 41（68.30） | 0.363 |
