# Supplementary material for: Early diagnosis and risk factors of diabetic peripheral neuropathy in type 1 diabetes: insights from current perception threshold testing
Source: Front Endocrinol (Lausanne). 2025 Mar 31;16:1496635. doi: 10.3389/fendo.2025.1496635 (PMC11994408; doi:10.3389/fendo.2025.1496635)
Supplement: Supplementary Table 2 — Comparison of CPT values between T1DM group and normal control group [P50 (P25, P75)]. A comparison with a healthy control group could interpret the data more accurately and help determine neurological dysfunction in DPN patients relative to non-diabetic patients, The significance level was set at α = 0.05, with P < 0.05 considered statistically significant. [file Table2.docx]

Table S2 Comparison of CPT values between T1DM group and normal control group [P50 (P25, P75)]

| Group | Left median nerve | | | Right median nerve | | |
| --- | --- | --- | --- | --- | --- | --- |
|  | 2000Hz | 250Hz | 5Hz | 2000Hz | 250Hz | 5Hz |
| T1DM group  （n=110） | 281  （206,320） | 125  （61,125） | 66  （66,99） | 281  （232,320） | 125  （125,146） | 89  （66,125） |
| Normal group（n=26） | 206  （198,232） | 80  （61,125） | 66  （43,89） | 232  （206,291） | 103  （75,146） | 43  （35,89） |
| P value | 0.025 | 0.012 | 0.006 | 0.008 | 0.027 | 0.000 |

| Group | Left sural nerve | | | Right sural nerve | | |
| --- | --- | --- | --- | --- | --- | --- |
|  | 2000Hz | 250Hz | 5Hz | 2000Hz | 250Hz | 5Hz |
| T1DM group  （n=110） | 470（350,510） | 189（146,211） | 103（66,148） | 470（350,526） | 189（146,255） | 120（66,170） |
| Normal group（n=26） | 350（300,479） | 125（101,189） | 81（66,120） | 375（350,479） | 125（101,167） | 74（66,102） |
| P value | 0.015 | 0.001 | 0.015 | 0.027 | 0.000 | 0.004 |

A comparison with a healthy control group could interpret the data more accurately and help determine neurological dysfunction in DPN patients relative to non-diabetic patients,The significance level was set at α = 0.05, with P < 0.05 considered statistically significant.
